# Investigating the nature and quality of locally commissioned evaluations of the NHS Vanguard programme: an evidence synthesis

**DOI:** 10.1186/s12961-021-00711-3

**Published:** 2021-04-12

**Authors:** Paul Wilson, Jenny Billings, Julie MacInnes, Rasa Mikelyte, Elizabeth Welch, Kath Checkland

**Affiliations:** 1 Centre for Primary Care and Health Services Research, University of Manchester, Manchester, UK; 2 Centre for Health Service Studies, University of Kent, Canterbury, UK

**Keywords:** Integrated care, National Health Service, Vanguard, New care models, Evidence synthesis

## Abstract

**Background:**

With innovation in service delivery increasingly viewed as crucial to the long-term sustainability of health systems, NHS England launched an ambitious new model of care (Vanguard) programme in 2015. Supported by a £350 million transformation fund, 50 Vanguard sites were to act as pilots for innovation in service delivery, to move quickly to change the way that services were delivered, breaking down barriers between sectors and improving the coordination and delivery of care.

**Methods:**

As part of a national evaluation of the Vanguard programme, we conducted an evidence synthesis to assess the nature and quality of locally commissioned evaluations. With access to a secure, online hub used by the Vanguard and other integrated care initiatives, two researchers retrieved any documents from a locally commissioned evaluation for inclusion. All identified documents were downloaded and logged, and details of the evaluators, questions, methodological approaches and limitations in design and/or reporting were extracted. As included evaluations varied in nature and type, a narrative synthesis was undertaken.

**Results:**

We identified a total of 115 separate reports relating to the locally commissioned evaluations. Five prominent issues relating to evaluation conduct were identified across included reports: use of logic models, number and type of evaluation questions posed, data sharing and information governance, methodological challenges and evaluation reporting in general. A combination of resource, data and time constraints means that evaluations often attempted to but did not fully address the wide range of questions posed by individual Vanguards.

**Conclusions:**

Significant investment was made in independent local evaluations of the Vanguard programme by NHS England. This synthesis represents the only comprehensive attempt to capture methodological learning and may serve as a key resource for researchers and policy-makers seeking to understand investigating large-scale system change, both within the NHS and internationally.

PROSPERO (Registration number: CRD42017069282).

**Supplementary Information:**

The online version contains supplementary material available at 10.1186/s12961-021-00711-3.

## Background

Globally across health systems there is a drive to roll out innovative models of care that will deliver better value for money and improve the quality of care. For example, in the United States, accountable care organizations have emerged as innovative payment and delivery models that aim to improve the care coordination and quality whist containing growth in healthcare costs [1]. A review examining integrated payment and delivery models in health and social care identified 38 schemes across eight countries but found that evidence for impact was weak [2, 3]. No scheme demonstrated sustained reductions in secondary care utilization, though there was some evidence that care could be shifted into the community and access to services could be improved [2]. Regardless, such schemes are increasingly viewed as crucial to the long-term sustainability of health systems, and how best to harness their potential in relation to the National Health Service (NHS) in England remains a concern of health policy [3, 4].

In England, the Health and Social Care Act 2012 made innovation in the provision of health services a statutory duty [5]. Further impetus for major system change was set out in the Five Year Forward View in 2014 which argued that the divide between primary care, secondary care, community services and social care was increasingly a barrier to the personalized and coordinated health services patients need [6].

With innovation in service delivery increasingly viewed as crucial to the long-term sustainability of health systems, NHS England launched the Vanguard programme in 2015 [7, 8]. Fifty Vanguard sites were to act as test beds for multicomponent innovations in service delivery (see Box 1), supported by a £329 million transformation fund from NHS England (https://www.england.nhs.uk/new-care-models/about/). NHS England also spent another £60 million supporting and monitoring the progress of Vanguards [9].

The overarching goal of the Vanguard programme was to enable local health economies to move quickly to change the way that services are delivered, breaking down barriers between sectors and improving the coordination and delivery of care. It is intended that the new models of care will improve: population health and well-being; quality and equality of care; and the overall health and care system efficiency. Five “new care models” were established (see Box 1).

The Vanguard programme represented a novel approach to NHS change and development. With no central blueprint for change, local Vanguards were intended to be locally driven test beds but ones that would be supported to transform their services and sustain the anticipated benefits over time. A national programme of support, costing £60 million over 3 years [9], sought to bring local teams together to share ideas, experiences and solutions to problems encountered.

The national support package was intended to accelerate implementation and to maximize the opportunities for spread [9]. Each Vanguard site received funding and support to move quickly to establish new ways of working. This included support to develop a “logic model” to guide initial development and provide a framework for describing the underlying assumptions between the proposed change(s) and the desired outcomes. In addition, NHS England allocated around £10 million to individual Vanguards to procure and fund a local evaluation from an independent evaluation partner(s). The expectation for local evaluation was that it would complement national interrogation of outcome metrics by examining the delivery of each Vanguard’s activities in depth [10, 11]. It was anticipated that local evaluations would:

- Capture and evaluate the transformation changes delivered by the Vanguards appropriately. Alongside knowing whether things have changed (through outcome metrics), it is important we understand how, and in what context, the changes have occurred.
- Understand the “reach” of the Vanguard locally. With this in mind, it is important to include output data such as the number of patients affected by changes made.
- Feed the information gathered into ongoing, on-the-ground delivery, so that services are continually improved.
- Share the learning gathered between the Vanguards and more widely, to promote replicability and scale up. Doing so will also help to ensure that we tackle any barriers/issues collectively, for the benefit of the whole.
- Embed a culture of evaluation and knowledge sharing within the Vanguard.

As part of the national evaluation of the New Models of Care Vanguard programme in England, we have conducted an evidence synthesis of the nature and quality of locally commissioned evaluations relating to three Vanguard types. These were enhanced health in care homes, the primary and acute care systems (PACS) and the multispecialty community providers (MCPs). Funding from NHS England for local evaluations amounted to around £7 million for these three types. Our synthesis focuses on describing what was commissioned and the methodological quality and completeness of reporting of each local evaluation.

Box 1. NHS Vanguard new care model types Integrated primary and acute care systems (PACS): Nine sites joining up general practice, hospital, community and mental health services

Enhanced health in care homes: Six sites offering older people better, joined-up health, care and rehabilitation services

Multispecialty community providers (MCPs): Fourteen sites focused on moving specialist outpatient and ambulatory care out of hospitals into the community

Urgent and emergency care: Eight sites developing new approaches to simplify and improve the coordination of services and reduce pressure on emergency departments

Acute care collaborations: Thirteen sites that link together local hospitals to improve their clinical and financial viability

## Methods

The review protocol was registered in PROSPERO (Registration number: CRD42017069282).

### Data sources and searches

In 2016, NHS England invested in the FutureNHS collaboration platform (Kahootz), a secure, online hub for the Vanguard and other integrated care initiatives. The platform was implemented as a direct response from Vanguards and other models of care for a place to work together more collaboratively. The repository was designated as the means by which Vanguards could store, share and access key documents in one central hub. Through this platform, registered users are able to access relevant information, documents and evaluation reports related to the Vanguard programme. Because of this, traditional review search strategies were not deemed necessary for the purpose of this synthesis.

Two researchers accessed Kahootz every month from June 2017 to September 2018 to search for documents relevant to the synthesis. At the start of 2018, the NHS England evaluation team had provided a spreadsheet of anticipated dates for the delivery of final evaluation reports which indicated most were expected to be delivered between April and June 2018. In September 2018, a cross-check of downloaded/extracted documents with the local evaluation deliverables spreadsheet revealed that a number of final reports were outstanding. Access to a shared folder on a restricted area of Kahootz was then given to the team by NHS England, and all available final reports and documents were downloaded. Any reports that may have been received after September 2018 are not included in the synthesis. In addition, to Kahootz, we also searched for eligible evaluation reports on Vanguard and named evaluator websites. Additional reports were received from local evaluation teams. All identified documents were downloaded, logged and stored in folders on a secure shared drive.

### Study selection

Any report or slide set from a locally commissioned evaluation of a Vanguard was eligible for inclusion. As our focus was on locally commissioned evaluations of Vanguards, any other external or national evaluations such as those conducted by the Health Foundation Improvement Analytics Unit were excluded from the review.

### Data extraction and quality assessment

For each identified evaluation, details of the evaluators, questions, methodological approaches and limitations in design and/or reporting were extracted and assessed by one researcher and checked by a second. Many Vanguards commissioned different evaluations to answer different questions. As there is no definitive checklist for assessing the quality of mixed methods evaluations, we adapted a set of quality questions originally proposed by O'Cathain et al. to assess the quality of mixed methods studies in health services research [12]. In the original development work for the checklist framework, the main quality issues identified were a lack of transparency for the mixed methods aspects of the studies and the individual components. It is now widely recognized that reporting is an important marker of quality [13], and underreporting can seriously distort the available evidence, compromise its usefulness and reliability, and may also mislead [13]. We used these questions as a guide to assess the overall approach to local evaluation taken in each Vanguard, including the appropriateness of the design, the transparency of reporting of the quantitative and qualitative components, and the extent which what was planned was delivered. The questions are:

- Is the quantitative component feasible?
- Is the qualitative component feasible?
- Is the mixed methods design feasible?
- Have both qualitative and quantitative components been completed?
- Were some quantitative methods planned but not executed?
- Were some qualitative methods planned but not executed?
- Did the mixed methods design work in practice?

### Method of synthesis

As the included evaluations were largely mixed methods with variation in the nature and type of quantitative, qualitative and cost components, we performed a narrative synthesis of the evidence. Consistent with an integrative approach to synthesizing evidence, this narrative synthesis aimed to present a descriptive summary of the nature, type and general quality of evaluations within, and then to generate, across Vanguard types, a number of themes relevant to the aims of this review. An iterative process of adaptation and refinement was undertaken by two researchers to generate initial themes. Themes were then discussed with the wider research team, refined and sense-checked against themes generated from the qualitative exploration of the experiences of evaluation leads undertaken as part of Work Package 1 of this national programme evaluation [14, 15]. This work conducted in parallel with the synthesis highlighted a number of challenges including perceived expectations, data access, availability and quality and evaluative timescales.

## Results

### Nature of local evaluations

We identified 115 local evaluation reports that were eligible for inclusion in this review. Thirty-two reports related to the local evaluations of the six enhanced health in care homes Vanguards. Each local evaluation is presented descriptively in Additional file 1 with any limitations in design and/or reporting highlighted. A total of 37 separate reports relating to the local evaluations of seven PACS Vanguards (19 of which were from North East Hampshire and Farnham) are presented in Additional file 2. Two Vanguards, Wirral and Salford, did not commission local evaluations via the new care models programme. Additional file 3 presents 46 separate reports relating to the local evaluations of nine MCP Vanguards. No local evaluations were commissioned by Calderdale or Lakeside Vanguards. Stockport did not receive evaluation funding as part of the new care models programme. Again, any limitations in design and/or reporting are highlighted. No reports were available for Fylde Coast or West Cheshire.

In addition to commissioning their own local activity, the five North East Vanguards (Gateshead [Enhanced Health in Care Homes], North East Urgent and Emergency Care Network [Urgent and Emergency Care Vanguard], Sunderland [MCP], Northumbria Foundation Group [Acute Care Collaboration Vanguard], Northumberland [PACS]), in collaboration with North East Commissioning Support, pooled resources to commission a regional evaluation. This evaluation was to explore regional implementation and aimed to identify key barriers and enablers and aspects of system transformation that could be shared across all Vanguard sites and which may be of wider interest to other regions in England. Details are presented in the Gateshead, Sunderland and Northumberland sections of Additional file 1, Additional file 2 and Additional file 3, respectively.

### Synthesis of findings

Five prominent issues relating to evaluation conduct were identified across included reports: use of logic models, number and type of evaluation questions posed, data sharing and information governance, methodological challenges and evaluation reporting in general. We describe each of these issues in turn.

### Use of logic models

All Vanguards were supported to produce logic models; a requirement of their funding in year 1 only. Logic models were to describe the anticipated inputs, outputs and impacts of the care model proposed. However, these proposed impacts are only partially reflected in the research questions proposed by the local evaluations. In the enhanced care home evaluations, only two (Nottingham and Sutton) explicitly reference logic models as part of their evaluation plans, with a third (Wakefield) including its logic model as an appendix. For PACS, only Morecambe Bay evaluators explicitly and consistently refer back to the logic model. In doing so, they emphasized a disconnect between what was specified and then actually delivered and highlight an apparent lack of a consensus on the ground in terms of what the Vanguard outcomes should be. Mid Notts stated that their evaluation sought to identify what impact the Vanguard programme was having on the outcomes outlined in the logic model, but this is not explicitly carried through to the findings. North East Hampshire and Farnham include the logic model as an appendix and state that where possible, logic models were used to develop the service evaluations conducted. For MCPs, the evaluators of Birmingham and Sandwell, Better Local Care (S Hampshire) and Principia all include reviews of Vanguard logic models as part of an analysis of programme documentation. Principia’s baseline assessment did flag up the need to continually update the Vanguard logic model and delivery plan to reflect the evolution of the programme, but whether this actually happened in the proposed phase 2 of the evaluation is unclear (as no detail on phase 2 is reported).

No other local evaluations either mentioned or related emergent findings back to the original logic model of the Vanguard. Logic models appear to have been used as a sense-making tool for initial programme development and not as an evaluative framework to assess whether the planned inputs and activities of each care model did lead to the anticipated outcomes. Any potential value to the evaluation process and as an ongoing programme management and improvement tool is not apparent in evaluation reports overall.

### Evaluation questions

Most Vanguards posed multiple questions to be addressed by the evaluator, and across the included evaluations we identified 184 evaluation questions across the Vanguards that commissioned local evaluators (see Additional file 4). The way many questions were framed was similar to those posed in the generic commissioning guidance circulated by NHS England. As a consequence, many questions lacked specificity and did not directly address key components of each Vanguard as espoused in the locally developed logic models. While many of the evaluations appear to address the research questions stated in the original commissioning briefs, others do not. Whilst some lack of consistency can be attributed to the iterative and formative nature of the evaluations conducted, with others it is less clear why there has been deviation from the original intentions. Some evaluation reports stated that they were addressing specific questions and then did not explicitly do so.

### Data sharing and other information governance issues

Despite significant efforts on the part of evaluators, a lack of data-sharing agreements and information governance procedures appear to have been significant barriers to data access and to the conduct of outcome analyses. Where data-sharing agreements were lacking and or there were data access issues, this significantly curtailed outcome analyses. For example, for the enhanced care home Vanguard Airedale, the initial local evaluation team was not able to access project metrics or routine service utilization or outcome data at all within the evaluation time frame. The evaluation team did, however, support the Vanguard in its Data Access Request Service application to NHS Digital. In the PACS, Harrogate, Isle of Wight and South Somerset all had difficulty accessing routine data, with a lack of data-sharing agreements and information governance constituting significant barriers to access. The challenge of obtaining data-sharing agreements was so significant for South Somerset that the evaluator was unable to undertake the quantitative analysis within the evaluation time frame. The activity analysis planned by Isle of Wight was not done, as they were unable to obtain a data-sharing agreement for analysis of social care data. Other data challenges included an absence of patient-level data for Morecambe Bay and Harrogate’s unsuccessful attempts to capture key service-level data. For the MCPs, significant data-sharing and information governance issues were experienced by the South Hampshire evaluators; local General Data Protection Regulation (GDPR) interpretation meant that Secondary Uses Service (SUS) data was unavailable to the evaluation team. Other data challenges included access to general practitioner (GP) appointment data on a borough-wide basis for Tower Hamlets, and Encompass was unable to link service user-level data to analyse the impact of the community hubs.

### Methodological challenges

Most Vanguards posed evaluation questions which to be addressed would necessitate qualitative, quantitative and economic methods. This is reflected in the approaches taken by the evaluators. For example, all care home evaluations were planned as mixed methods evaluations or perhaps more accurately, planned to utilize a mix of methods. More so than the PACS or MCP Vanguards, the care home Vanguards all involved the rollout/spread of defined interventions. Given this, there was an opportunity to generate generalizable knowledge through the conduct of natural experiments that evaluated the longitudinal effects of intervention implementation. Only the regional funded evaluation of the five North East Vanguards attempted a quasi-experimental design in the form of interrupted time series (ITS). Instead, evaluations largely focused on describing the implementation context and the organization and delivery of care as it changed, conducting “before and after” outcome analyses on specified utilization and performance metrics and made attempts to capture stakeholder reflections and experiences of the Vanguard.

With quantitative evaluation, the pursuit of the counterfactual proved challenging with most struggling to create meaningful comparators. In the enhanced care homes, one particular issue was the identification of care home residents themselves. As care homes do not have a unique reference number, analyses used the postcode of the care home as a proxy indicator to identify residents and their associated outcomes. Doing so increased the risk of bias in terms of overestimation of the impact on outcomes as data may include non-care home residents (who could be subject to other confounders) who share a postcode. Sutton evaluators were unable to include a comparator as originally planned and instead devised a weighting approach to enable comparison across care homes. Although this approach was novel and would have provided some insight into impact for the local audience, it lacks external validity and has limited generalizability. In the PACS, Harrogate evaluators failed to create a meaningful comparator for any of their planned analyses. North East Hampshire and Farnham, Isle of Wight and Morecambe Bay also did not appear to have attempted comparative evaluation, opting instead for before and after activity analyses. For MCPs, Sunderland and West Wakefield do not appear to have been attempted comparative evaluations. Principia planned to compare local activity against national trends, but no report is available of that phase. Encompass evaluators stated that resources needed to generate a sufficiently meaningful comparator were beyond the resources and time frame set for the evaluation.

Where local sources of routine data have been available, completeness and accuracy of data sets has been a significant issue. In several instances, secondary analyses were constrained by time required for data cleaning and accounting for missing data. Four of the PACS vanguards (Isle of Wight, Morecambe Bay, Northumberland and South Somerset) planned economic analyses that were subsequently not realized.

With the qualitative components of evaluations, many evaluators appear to have experienced some challenges in engaging participation from patients, service users and indeed staff with low numbers of interviews and survey response rates a feature across evaluations (indeed where numbers are reported). Good qualitative research offers explanatory power and nuanced insight [16–18]. The qualitative aspects are largely descriptive lists with no real attempt to theorize, generate themes or to integrate findings with other data sources. There are some instances where evaluators describe use of specific approaches such as normalization process theory, but without more detailed reporting it was difficult to gauge whether these approaches were really applied beyond the superficial. Planned sampling methods and sizes, the approaches taken, nonparticipation rates and methods of analysis are all not well reported across evaluations.

### Reporting

No standardized reporting requirements were proposed at the outset of the Vanguard programme, and as a consequence, many of the local evaluation reports are lengthy and challenging to navigate. Lack of standardized reporting makes it difficult to identify the methods used, and findings are often not linked back to the original research questions proposed. For the quantitative components, detail is often lacking on the planned statistical approach for analysis, though this is often implicit in the presentation of results. As mentioned above, the qualitative aspects of many evaluations are often very poorly reported, making it difficult to assess execution. Sutton, Harrogate, Morecambe Bay, Dudley, Encompass and the regional North East evaluation are all clearly reported evaluations. Morecambe Bay does attempt to relate findings to the original intentions of the Vanguard. Harrogate’s evaluation, although severely curtailed, does attempt to situate tentative findings in relation to some existing evidence for intermediate care and integrated services. Encompass is one of the few evaluations to employ a theoretical framework (evidence integration triangle) and undertakes an overarching synthesis that explicitly relates back to the research questions originally posed.

### Assessing the success of execution of local evaluations

Tables 1, 2 and 3 present a summary of our assessment of the success of execution of each local evaluation by Vanguard type. We have used the questions posed by O'Cathain et al. [12] to assess the appropriateness of design, transparency of reporting of the quantitative and qualitative components, and the extent which what was planned was delivered. Where data-sharing agreements were lacking and or data access issues significantly curtailed prespecified analyses, we have classified the quantitative components as not feasible. In the enhanced care homes, planned quantitative analyses were either modified or not conducted. Although not without some operational challenges and acknowledged limitations, the regional North East evaluation would appear to represent the most coherent attempt to generate generalizable knowledge beyond their own setting. Of the others, the MCP Vanguard Encompass was one of the few evaluations to employ a theoretical framework (evidence integration triangle).

**Table 1:** Assessment of the success of execution of local evaluations for the enhanced care home Vanguards (adapted from O'Cathain et al. [12])

	Airedale	Gateshead	Herts	Notts	Sutton	Wakefield
Quantitative component feasible?	No	Yes	No	Yes	Yes	No
Qualitative component feasible?	Yes	Yes	Yes	Yes	Yes	Yes
Mixed methods design feasible?	No	Yes	No	Yes	Yes	No
Have both components been completed?	No	Yes	No	Unclear	Yes	No
Quantitative methods planned, not executed?	Yes	Yes	Yes	Unclear	Yes	Yes
Qualitative methods planned, not executed?	Yes	No	No	Unclear	Unclear	No
Did mixed methods design work in practice?	No	Yes	No	Unclear	Yes	No

**Table 2:** Assessment of the success of execution of local evaluations for PACS Vanguards (adapted from O'Cathain et al. [12])

	Harrogate	NE Hants	IoW	Morecambe	Northumb	Mid Notts	S Somerset
Quantitative component feasible?	Yes	Yes	Yes	Yes	Yes	Yes	No
Qualitative component feasible?	Yes	Yes	Yes	Yes	Yes	Yes	Yes
Mixed methods design feasible?	Yes	Yes	Yes	Yes	Yes	Yes	No
Have both components been completed?	No	Yes	No	No	Yes	Yes	No
Quantitative methods planned, not executed?	Yes	No	Yes	Yes	Yes	Unclear	Yes
Qualitative methods planned, not executed?	Yes	No	Yes	Yes	No	Unclear	Yes
Did mixed methods design work in practice?	No	No	No	Yes	Yes	No	No

No local evaluations commissioned by Salford or Wirral Vanguards via the new care models programme

**Table 3:** Assessment of the success of execution of local evaluations for MCP Vanguards (adapted from O'Cathain et al. [12])

	BLC	Birmingham	Dudley	Encompass	Erewash	S Notts	Sunderland	T Hamlets	W Wakefield
Quantitative component feasible?	No	N/A	Yes	Yes	Yes	Yes	Yes	Yes	Yes
Qualitative component feasible?	Yes	Yes	Yes	Yes	Yes	Yes	Yes	Yes	Yes
Mixed methods design feasible?	No	Yes	N/A	Yes	Yes	Yes	Yes	Yes	Yes
Have both components been completed?	No	Yes	N/A	Yes	No	Unclear	Yes	No	Yes
Quantitative methods planned, not executed?	Yes	N/A	Unclear	Yes	Unclear	Unclear	No	Yes	No
Qualitative methods planned, not executed?	No	No	Unclear	Yes	Yes	Unclear	No	Unclear	No
Did mixed methods design work in practice?	No	Yes	N/A	Yes	No	Unclear	Yes	No	Yes

No local evaluations commissioned by Calderdale or Lakeside Vanguards. No details available for Fylde Coast, Stockport or West Cheshire. Sunderland assessment based on the North East Vanguard evaluation

## Discussion

Innovation in health service delivery without adequate evaluation can lead to misattribution of effects and worse, the wider adoption of ways of working without proven benefits over existing alternatives [19]. Independent local evaluation was a key pillar of the evaluation plan for the new care model Vanguard programme. NHS England made significant resources available to individual Vanguards to procure and fund a local evaluation from an independent evaluation partner(s). This review represents the first attempt to systematically assess the nature and quality of the evaluations commissioned and to capture methodological learning to inform future endeavours of this type. The synthesis summarizes a significant grey literature of local evaluation reports, not all of which are publicly available. By summarizing this evidence, we have ensured that the reports continue to be publicly available. This review therefore represents the only comprehensive mapping of what was commissioned and conducted and may serve as a key resource for researchers and policy-makers, both within the NHS and internationally.

### Limitations

Whilst we have included 115 reports from local evaluations in this review. It is possible that we have not identified some or that additional reports may have been submitted to the NHS England evaluation team after our synthesis was complete. We are also aware that evaluators have fed back their learning using means other than reports including slide sets, webinars and face-to-face interactions. Despite this, we think it unlikely that any unidentified evaluations will be significantly different to those included in this review. Nor do we think the key themes we have identified would differ significantly had we been able to comprehensively capture other modes of communication used by evaluators.

Poor reporting practices can seriously distort the available body of evidence and compromise its usefulness and reliability [20]. There is no definitive checklist for assessing the quality of mixed methods evaluations, and so there are some limitations with the approach we have employed. There is a degree of subjectivity in our assessments of the feasibility and appropriateness of each evaluation design. Reporting is as important a part of an evaluation as its design or analysis [13], and our assessments were often hampered by a lack of methodological specificity in evaluation reports, making it difficult to make judgements about the extent to which individual components were either feasible or indeed realized. The criteria proposed by O'Cathain et al. at least provides a structure for assessing the feasibility, appropriateness and overall quality of evaluation design across Vanguards [12].

Although a significant amount of money was spent on commissioning and conducting multiple local evaluations, each was relatively small-scale and context-specific. It is perhaps unsurprising that the regionally funded evaluation of the five North East Vanguards represents the most coherent attempt to address a wide range of questions through use of rigorous and transparent methods [21]. Each individual North East Vanguard also commissioned additional small-scale evaluations to explore aspects deemed locally important, but that fell outside of the regional evaluation. This suggests that NHS England’s recognition of the need for “local evaluation for local people” was right but that achieving the balance between delivering a detailed understanding of what was working, why and how in each context [10, 11] and formative insight to shape local implementation was harder to operationalize in practice.

In keeping with other efforts to evaluate large-scale system change [22, 23], mixed methods were widely adopted to understand the nature of change efforts and how change occurred. Future evaluations of large-scale service change should continue to consider such a multifaceted approach but perhaps with less prescription of what should be explored in depth at the local level. In the evaluation of the New Models of Care Vanguard programme, the ability to deliver robust counterfactual analysis has been limited to the national-level evaluation teams [24–26]. Given the apparent challenges faced by local evaluation teams in trying to deliver counterfactuals (under resource constraints and limited time horizons), there is an argument that local analysis should focus on providing causal explanations for impact in a given context. Less local prescription may also be warranted for formative evaluation, exploration of local patient experience and/or on capturing the costs of local change. The relative success of the combined North East evaluation in surfacing common barriers and enablers of system change suggests a meso level of analysis through which generalizable knowledge can be generated. The aims of any evaluation strategy of course need to balance what is desirable with what is actually achievable within available time and resource constraints. Our synthesis highlights a number of common issues across the local evaluations. We summarize our recommendations for each of these as follows:

### Research questions

Local evaluations were expected to generate evidence that would inform the main national evaluation questions set out by NHS England [10, 11]. Although originally issued as guidance, there is some evidence of a “lift and shift” approach to local question formulation with very literal use of the questions circulated by NHS England. This “lift and shift” may be rooted in the national emphasis on examining the entirety of a Vanguard’s programme of activity. This meant that many Vanguards were often asking for an “evaluation of everything” when a more specified approach to question formulation may have led to more focused/meaningful exploration of the specific interventions/initiatives of the local Vanguards. The issue may have occurred because local Vanguard teams issuing tenders for the evaluation did not have specialist understanding of research/evaluation methodologies. It may therefore be beneficial for evaluators to be funded for a pre-evaluation stage [27]. Doing so may not only aid the development of evaluation questions that meet local expectations and national requirements, but also provide an opportunity to assess whether these can be addressed via locally available data and collection processes.

### Data sharing and other governance issues

Data-sharing agreements and information governance more broadly posed a significant barrier to obtaining relevant data (this was especially, but not exclusively, true for the quantitative part of the analysis). This has significant implications for future evaluation programmes of this type. Indeed, it can be argued that without data-sharing agreements in place from the outset, many plans for quantitative analysis were not feasible. Interviews with evaluation leads further supported this finding and emphasized that even where solutions were eventually found, information governance procedures created significant delays that subsequently compromised the feasibility of original plans [14, 15]. Given this, the responsibility for data access may best lie with those specifying and then commissioning evaluations. Information governance agreements should be a priori established before the evaluation commences whenever possible; alternatively, evaluation plans should consider scenarios where data sharing cannot be established in determining what can be feasibly achieved.

### Counterfactuals

Many of the evaluations could not obtain a suitable counterfactual. The data synthesis found that few local evaluations had the time, resources or skill sets to conduct comparative impact assessments on improvements to the quality and efficiency of care, while interviews with evaluation leads demonstrated that most interviewees did not see the counterfactual as useful and favoured other comparative methods. Obtaining a counterfactual may not be possible for local evaluations and may be best placed with national-level evaluations instead.

### Quality of research methods

Evaluation leads often focused on the challenges associated with quantitative aspects of the evaluation. However, the evaluation synthesis highlighted that qualitative methods were often poorly executed (or written up in a way that suggests this). Many of the local evaluations did not offer explanations and/or nuanced insights into the Vanguard operation, and did not integrate with other data sources to explain or enhance the credibility of the findings. However, there were difficulties in gaining access to data and in engaging professionals and service users as research participants which may have limited the depth of analysis. How evaluations using both qualitative and quantitative methods will integrate findings from both approaches should be clearly communicated from the outset.

### Reporting in general

A key expectation of local evaluations was that efforts would be made to share local learning both between the Vanguards and more widely, to promote replicability and scale up. As with research generally, it is crucial that evaluators provide sufficient detail on their methods and the relationship between the analysis and the findings in the report so that readers can assess the credibility of the findings. No standardized reporting requirements were proposed at the outset of the Vanguard programme, and as a consequence, many of the local evaluation reports are lengthy and challenging to navigate. This lack of standardized reporting has made it difficult to identify the methods used and to describe the key findings in relation to the questions posed. Poor reporting has been a barrier to learning in previous innovation programmes [28]. We therefore argue that a more consistent reporting style would have made the reports much more accessible and improved clarity on the methods used in the evaluations, thus ensuring that learning is systematically captured in a generalizable format.

## Conclusions

The Vanguard programme was conceived as a series of locally driven attempts to transform and integrate health and care services that would contribute to the development of care model prototypes that could later be replicated rapidly across the rest of England. Significant investment was made in support and evaluation of each Vanguard by NHS England. This synthesis represents the only comprehensive attempt to capture methodological learning and may serve as a key resource for researchers and policy-makers seeking to understand investigating large-scale system change, both within the NHS and internationally.

## Supplementary Information

**Additional file 1.** Nature of local evaluations for enhanced care home Vanguards

**Additional file 2.** Nature of local evaluations for PACS Vanguards

**Additional file 3.** Nature of local evaluations for MCP Vanguards

**Additional file 4.** Local evaluation questions

## Data Availability

All available data can be obtained from the corresponding author upon reasonable request.

## Abbreviations

MCP: Multispecialty community provider
NHS: National Health Service
PACS: Integrated primary and acute care systems
