# Supplementary material for: Investigating the nature and quality of locally commissioned evaluations of the NHS Vanguard programme: an evidence synthesis
Source: Health Res Policy Syst. 2021 Apr 12;19:63. doi: 10.1186/s12961-021-00711-3 (PMC8042862; doi:10.1186/s12961-021-00711-3)
Supplement: Supplementary file 1 — Additional file 1. Nature of local evaluations for enhanced care home Vanguards [file 12961_2021_711_MOESM1_ESM.docx]

**Additional File 1 - Nature of local evaluations for the Enhanced Care Home Vanguards**

| Vanguard / Evaluator | Design | Limitations |
| --- | --- | --- |
| Airedale  Yorkshire and Humber AHSN Improvement Academy  York Health Economics Consortium (YHEC)  (4 reports) | Originally conceived as a mixed-methods evaluation involving retrospective before-and-after design with controlled comparisons.  Qualitative component focused on stakeholder reflections conducted post-intervention, using individual interviews, focus group interviews and online or paper surveys  YHEC conducted second attempt at assessing impact of telemedicine service using retrospective before-and-after design with controlled comparisons. Including analysis by different CCG, type of home (residential v nursing), service usage, environment (urban v rural).  YHEC also developed a return on investment analyses for the overall programme and the sub-levels identified above. | Significant data sharing and information governance issues – AHSN unable to access Airedale project metrics, utilisation data or outcome data.  Exact number of care homes receiving telemedicine service unclear. Initially 248 stated but then 235 (148 installed pre- and 87 during Vanguard period) 41 also de-installed. A further 50 homes did not receive the service.  YHEC evaluation based on data relating to 141 homes and 25 controls. Analysis based on a data set collated by an unnamed business intelligence organisation. Data cleaning revealed a large number of anomalies and missing data. Cleaned data set was reduced from 290,000 data points to 48,000 for the analysis. |
| Gateshead  NE Vanguard evaluation:  Institute of Health and Society, Newcastle University /  Centre for Public Policy and Health, Durham University /  Health and Life Sciences, Northumbria University  Firefly Research /  Institute of Health and Society, Newcastle University  University of Sunderland  (5 reports) | Combined NE Vanguard evaluation mixed-methods design, combining qualitative and quantitative approaches, to provide contextual understanding of the organisational, technological and economic facilitators and barriers shaping the implementation of the Vanguards programme. Evaluation conducted in three phases: (1) in-depth review of local documentation, semi-structured interviews with key stakeholders involved in the implementation of each Vanguard to identify organisational and technological enablers and barriers; (2) quantitative analysis and economic evaluation; (3) overarching synthesis and identification of emerging key messages for shared learning.  Quantitative analysis was conducted on relevant outcomes/performance metrics for care homes obtained from the Vanguard team. Analysis utilised ITS and Cox’s regression in order to make inferences regarding outcomes. The analysis conducted separately in each locality because of different stages of rollout. The cost analysis involved an estimate of the costs of running the Vanguards /the economic impact of the Vanguard on the key performance metrics provided.  17 interviews, conducted with senior managers and IT managers involved in the implementation of the Vanguard, to explore perceptions and experiences of the programmes’ processes, outcomes and impact. Transcribed interview data and fieldwork notes were analysed using thematic analysis to generate category systems and repeated themes from a regional perspective.  Firefly/ IHS qualitative evaluation involved semi structured interviews with 23 stakeholders, including 13 Pathway of Care (PoC) members Interviews supplemented with observations of Pathway of Care meetings. Stakeholders directly and indirectly involved were interviewed, to capture a broad range of perceptions of the PoC. Data analysed using Framework approach.  Northumbria NEWS evaluation: quantitative analysis of NEWS scores to assess the relationship between this score and other demographic and clinical data; qualitative exploration of professionals’ (n=13) decisions relating to admissions to hospital and experiences of NEWS within the context of care homes  Sunderland evaluation (informed by ‘realistic’ evaluation) involved semi structured interviews with 11 (of 29) informants directly involved in the contracts and commissioning work stream. | Data collection used post-code areas around care homes - possible overestimation of the impact on outcomes. Limited data points available for the post Vanguard period - potential seasonal effects were not adjusted for.  NEWS - small number of homes took part in the pilot (n=2 out of planned 6). Complete data for 84 residents only.  Sunderland evaluation not a realist evaluation despite being labelled as such.. |
| East and North Hertfordshire  Centre for Research in Primary and Community Care, University of Hertfordshire  (3 reports) | Secondary analysis of quantitative data derived from two sources: local Trust and SUS. Descriptive statistics were used to build a picture of HomeFirst and Stroke ESD users and service use.  For HomeFirst, analysis explored impact on relevant outcome (service utilisation) against matched controls. No matched controls possible for Stroke ESD.  Qualitative component involved semi structured interviews with senior and team managers (n=10), frontline staff (n=31) and patients and carers (n=9). Data relating to staff visits was obtained from patient care notes. A focus group was undertaken with GPs and practice nurses, and an online survey on integration (administered via SurveyMonkey) was circulated to staff via team managers. Data analysis was informed by a 'patient-centric' framework proposed by Kodner and Spreeuwenberg (2002).  Cost comparison with usual service use based on unit cost data was planned but data was deemed insufficient in detail to be able to conduct a meaningful analysis. | Data sharing and information governance issues - Delayed access to local Trust and SUS data. Data provided via intermediary (MedeAnalytics) incomplete Secondary analysis constrained by time and missing data.  Used matched controls based on gender an age but unable to match health status |
| Nottingham City  Experience Led Care / Age UK  Cordis Bright  (11 reports) | Surveys undertaken by ELC/ Age UK in 5 care homes (2015/2016) and 5 care homes (2017/2018). Both sought to understand the views and experiences of residents and consultees in relation to living in a care home. 50 respondents took part in each study, made up residents who participated independently or were supported by a consultee.  ELC commissioned to review the results of the Age UK Notts consultation. ELC then conducted baseline evaluation of resident and care home staff experience of living and working in 5 care homes (2016/17). Purposive and convenience sampling produced a sample of 50 residents (20 supported by a consultee) and 50 staff.  Cordis Bright commissioned to undertake a process and impact evaluation of the Vanguard. Baseline evaluation involved in-depth review of local and national documentation and a review of relevant local data provided by Vanguard. Semi-structured telephone interviews with 8 key stakeholders involved in the design and/or implementation of the Vanguard were also conducted. A review of literature relating to ‘what works’ in delivering assistive technology and clinical pharmacy in care homes was undertaken.  Impact or summative evaluation following principles of action research was conducted in a series of stages. Telephone interviews with 31/36 key stakeholders with insight into the design, implementation and impact of the Vanguard. Visits to six care homes including full-day visits to two homes operating the telemedicine system. Interviews with 8 professionals working in care homes to understand their experiences. Online survey of care home managers and staff and staff from CCG, local authority and the wider health and social care system (38 usable responses from 62 returns - unclear denominator). Review of strategic and operational documentation and data provided by the CCG. A review of the clinical pharmacy work stream focused on benchmarking practice against local, BNF and NICE guidance and to assess impact of the work stream. Included two workshops with the clinical pharmacists to understand how the service is operating, its strengths, and where it might improve. Analysis of care home level data using data from NHS Nottingham University Hospitals Trust (NUH) and East Midlands Ambulance service (EMAS). The CCG provided aggregated data from NUH from January 2016 to January 2018 on non-elective admissions and LoS for each care home in the Vanguard also provided aggregated data from EMAS for January 2016 to December 2017 on number of ambulance call-outs and ambulance conveyances. Comparative analysis between frequent users of telemedicine or high engagers with clinical pharmacy care homes that have received neither telemedicine nor clinical pharmacy reviews. Using price tariffs a cost avoidance figure was calculated. This formed the basis of a return on investment calculation, based on budget data provided by Nottingham City CCG. | Cordis Bright literature review review of academic and grey literature, described as designed to establish “what works” but non-systematic and restricted to Google search only, Only details of search terms presented and no detail on review processes.  Summative evaluation unable to access data on secondary care service use at a resident level (so care home level instead).  Challenges in gathering patient/ carer experiences  Not details on sampling or key informant identification presented.  Number of care homes on which the Vanguard could impact was much smaller than anticipated reducing ability to assess City-wide outcomes. |
| Sutton  SQW / SCIE  (5 reports) | Mixed methods evaluation. Baseline evaluation involved in-depth review of local documentation and local data relating to the Vanguard.  Quantitative analysis was conducted on relevant outcomes/performance metrics derived from Local Trust and London Ambulance Service data. In absence of counterfactual, analysis used a weighting approach - each intervention was assigned a number of points based on the likelihood of it contributing to a change in outcomes. The points for all the interventions for each care home were then added together to give a final score. Outcome data then compared for care homes according to their final score to ascertain if care homes with a higher score experienced greater improvements.  Qualitative component involved semi structured interviews with those involved in support/delivery of the Vanguard (n=14) to gather views on what is working well/not well, for whom and why. Supplemented with survey of care home staff (34 respondents, no denominator) SCIE conducted interviews with family/friend/ carers (n=11) from homes with moderate to significant engagement to explore impact, the ‘active ingredients’ and unintended costs/ consequences. Supplemented with survey (N=5). | Data used post-code areas around care homes - possible overestimation of the impact on outcomes  Did not include an assessment of a counterfactual as was originally planned.  Discrepancies between local Trust and LAS data (conveyances and A&E attendances)  Unable to conduct planned analyses using local data for ASCOT, AQP or for the link nurse intervention (paper records only) |
| Wakefield  Wakefield Public Health Intelligence  HealthWatch / Niche Health and Social Care Consulting  (4 reports) | Originally conceived as a before-and-after design comparing a random sample from intervention with matched controls. Unable to do so revised design to use the care home as the unit of study, with postcode as a proxy indicator for intervention. Statistical process control charts used to identify significant change in service utilisation.  HealthWatch survey included a selection of National Voices ‘I’ statements to measure the variety and integration of services going into the care homes; a validated wellbeing measure (ONS-4); and two qualitative questions regarding independence and wellbeing. Purposive sampling identified 5 care homes to take part in the baseline and follow up (1 Yr) surveys (only 3 care homes were included in Follow up) 42 resident survey interviews in May/June 2016 and 32 follow up interviews in February 2017. | Unable to find suitable comparator (postcode proxy used)  Lack of data sharing data sharing and information governance issues meant Public Health unable to gain access to SUS (local Trust data used instead but significant data anomalies)  SPC charts of change in unplanned admissions used 2 Sigma rather than standard 3 Sigma  Unable to establish patient level linked dataset so unable to determine which intervention elements had most impact.  Follow up survey data from 3/5 care homes |
