# Supplementary material for: Investigating the nature and quality of locally commissioned evaluations of the NHS Vanguard programme: an evidence synthesis
Source: Health Res Policy Syst. 2021 Apr 12;19:63. doi: 10.1186/s12961-021-00711-3 (PMC8042862; doi:10.1186/s12961-021-00711-3)
Supplement: Supplementary file 2 — Additional file 2. Nature of local evaluations for PACS Vanguards [file 12961_2021_711_MOESM2_ESM.docx]

**Additional File 2 - Nature of local evaluations for the PACs Vanguards**

| Vanguard / Evaluator | Design | Limitations |
| --- | --- | --- |
| Harrogate  NIHR CLAHRC Yorkshire and Humber  School of Health and Related Research, University of Sheffield; Centre for Health Economics, University of York;  Sheffield Hallam University;  York Health Economics Consortium.  (3 Reports) | Three work streams: 1) Theory-led qualitative evaluation, 2) Development of Evaluation & Monitoring Metrics, 3) Economic Evaluation.  WS1 = Literature review to identify a range of intervention methods for systems change.  Documentary analysis: Retrospective documentary analysis to develop a timeline and thematic understanding of the process changes and reporting. Interviews with 24 service managers. Interviews with four GP staff and Practice management involved in the Integrated Response Service (IRS). Two workshops each with a group of six members of the 'pop-up' IRS team, to explore what was working well, what could be improved and discuss potential alternative strategies. Semi-structured telephone interviews with up to 10 patients to understand experiences of using the new integrated service.  WS2 = Participant observation conducted during weekly Steering Group meetings. Analysis of internal documents: Informal interviews and meetings: with the members of the management team to elicit requirements for the dashboard.  Review of the identification and use of data to provide suggestions for the choice and representation of metrics. Assessment of the utility and acceptability of various dashboard platforms for visual representation of data.  Development of a Service-User-Record (SUR) to capture key service-level data, which was not successfully implemented.  Workforce Dynamics questionnaire (WDQ) used to survey the functioning of the interdisciplinary team.  WS3 = ITS analysis on key performance metrics derived from SUS data conducted using Stata 14 using the Cumby-Huizinga general test for autocorrelation. | Access to data limited by a number of factors -challenges of information governance, the excessive pressures on provider information teams, and the lack of means to identify patients who have interacted with the service. Evaluation restricted to aggregated data for metrics covering activity levels in Local Trust.  *Large level of uncertainty across the ITS analyses as a result of insensitivity of the data available.* Comparator evidence unavailable for all metrics.  Attempts to initiate service-level measurement tools did not result in systematic or complete data collection. Service-User-Record to capture key service-level data was not successfully implemented.  Patient experience interviews not conducted. |
| North East Hampshire and Farnham  Wessex AHSN;  Centre for Implementation  Science, University of Southampton.  (19 Reports) | A programme of evaluation covering 23 separate services conducted over two years. Common set of methods used across evaluations:  R-Outcomes measure (a validated short generic PROM) used by Wessex AHSN) used to measure patient and staff experience. 530 staff outcomes received and analysed across 6 collection periods over 2 years (n=?). 3,300 baseline and follow-up patient responses across services over 2 years (n=?)  Staff (n=51) and patient (n=62) interviews to understand experiences and challenges of implementation and nature and extent of change across services. Supplemented by patient survey (n=193).  Team observation focus groups (n=80) and survey (n=137) of integrated care teams using NOMAD questionnaire (Normalisation Process Theory) to understand the extent to which teams were able to embed new care model.  Thematic analysis of 124 case studies. Collected by staff using short template to describe the circumstances, intervention and outcomes for 8-10 of their patients.  Modelling evidence of impact on activity levels over time to estimate potential system savings. Comparison with costs to identify a potential return on investment. Before and after activity analysis undertaken by CSU using pseudo-anonymised patient records to measure the impact of new care models on principally emergency admissions. | Overall, programme of evaluation limited through decision to undertake series of small scale evaluations.  Modelling of impact on activity levels based on very small numbers of patients over short time frames (3 months).  Cumulative R-Outcomes responses large but small at service level.  Limited reporting of qualitative elements across services; small numbers of participants over 23 services.  Application of NOMAD questionnaire based on small numbers at service level. Unclear extent of team observation (single observation of MDT meeting or at ‘away day’ rather than routine and longitudinal). |
| Isle of Wight  Wessex AHSN  (5 Reports) | Case management: focus group discussions with MDT coordinators and the NOMAD questionnaire employing Normalisation Process Theory (NPT) (to focus group participants). Semi-structured interviews with programme leads, GP leads and specialist roles to understand implementation issues (along NPT lines) and gather views on the perceived effects of the MDT process  Local Area Coordination: staff interviews to understand the experiences, challenges, implementation and effects of LAC work.  ‘People’ interviews to understand people’s general experience of receiving LAC support.  Case studies provided by LAC staff to explore situations, processes and outcomes of LAC work.  Survey of professionals to explore the experience, process, implementation, effects, and challenges. Data from R-Outcomes measures (validated short generic PROM) being used by Wessex AHSN). Data was captured at service entry point and at approximately eight to 10 weeks.  Care Navigator staff interviews to understand the experiences, challenges and CN implementation.  ‘Person’ interviews to understand people’s general experience of receiving CN support.  Case studies provided to compliment other qualitative and quantitative data collected. Staff survey to understand general experience of CNs. Data from R-Outcomes measures captured at CN entry point and at completion?  Economic assessment of impact of CN service on the use of health and social care services and to calculate potential returns on investment for a number of scenarios. | CM: Limited number of participants. Three MDT coordinators participated in focus group and five staff from GP surgeries adopting CMoTAR were interviewed.  Evaluators state no ability to directly select participants to attend the focus group, or participate in interviews. Not possible to recruit staff from surgeries that did not adopt CMoTAR. No details on sampling strategy provided.  LAC: No sampling or recruitment detail provided for staff interviews (n=12) or ‘people’ interviews (n=9).No detail on selection or who completed the 22 case studies. No denominator for survey (n=23) or R-Outcomes measures (n= 40 service entry, n=45 for follow up).  CN: No sampling or recruitment detail provided for staff interviews (n=8) or ‘people’ interviews (n=7).No detail on selection or who completed the 11 case studies. 24/32 eligible participants completed survey. No denominator for survey (n=23) or R-Outcomes measures (n= 117 service entry, n=121 for follow up).  Planned economic analysis not done. Lack of availability of activity data. Unable to get data sharing agreement for analysis of social care data. |
| Morecambe Bay  Health and Social Care Evaluations, University of Cumbria,  (3 Reports) | Realist evaluation originally comprising four elements: 1. Desk-based study of the geographical, demographic and cultural contexts of the Vanguard. 2. Semi-structured interviews with 54 service providers and focus groups with 34 patients to gather perspectives around what changes, and who is affected; specifically, around population-based approaches, quality and integration of care, changes in culture and behaviour, and distribution of resources. 3. A large-scale survey of service providers and users to provide a broader set of qualitative and quantitative measures to test the evaluation hypotheses. 4. Assessment of the economic benefits of the Vanguard to identify changes in resource use and cost for specific interventions and evaluating the Vanguard’s overall performance against metrics developed for the Morecambe Bay Accountable Care System Integrated Performance Report (Elective Care =outpatient first attendances and follow-up; Out of Hospital = A&E attendances, non-elective admissions, non-elective bed days and ward closures).  The year 2 phase of evaluation to focus upon three pathways (respiratory, paediatrics and frailty) in three integrated care communities (ICCs) (Barrow Town, Bay and East) revised to focus on respiratory across all ICCs. Evaluation comprised 37 (17 conducted) interviews with staff involved in the development, leadership and delivery of the respiratory pathway. In-depth qualitative interviews with patients on experiences of care and the perceived impact of pathway. Observation of MDTs (yet to commence). Economic impact assessment to assess cost-effectiveness of the new way of working. | Lack of consensus about what ‘what success looks like’ and lack of overarching evaluation strategy on part of Vanguard.  Main data challenge was developmental nature of Vanguard - interventions targeted relatively small populations and required dataset would be at patient level. Difficult to obtain any data at this level.  Large scale survey not delivered (n=13 responses).  Data availability issues meant not able to include cost inputs in the economic analysis.  Year 2 assessment of the economic benefits of the Vanguard not undertaken.  Year 2 patient interviews postponed as too few patients had experienced pathway.  Overall realist approach only partially delivered and undermined by lack of a coherent programme theory (from the Vanguard’s perspective) from the outset |
| Northumberland  NE Vanguard evaluation:  Institute of Health and Society, Newcastle University /  Centre for Public Policy and Health, Durham University /  Health and Life Sciences, Northumberland University  (2 Reports) | Combined NE Vanguard evaluation mixed-methods design, combining qualitative and quantitative approaches, to provide contextual understanding of the organisational, technological and economic facilitators and barriers shaping the implementation of the Vanguards programme. Evaluation conducted in three phases: (1) in-depth review of local documentation, semi-structured interviews with key stakeholders involved in the implementation of each Vanguard to identify organisational and technological enablers and barriers; (2) quantitative analysis and economic evaluation; (3) overarching synthesis and identification of emerging key messages for shared learning.  Quantitative analysis was conducted on A&E attendances and emergency admissions for all Northumberland CCG patients from April 2014 to December 2016. (14-months pre and 21-months post). The performance data was split further by hospital Trust in order to view those patients from Northumberland who attended emergency care provision outside of the Northumberland area. Analysis utilised ITS and Cox’s regression in order to make inferences regarding outcomes  The cost analysis involved an estimate of the economic impact of the Vanguard on the key performance metrics provided. Despite requesting data regarding the cost of Vanguard provision for the PACS, the evaluation team did not receive this.  13 interviews, conducted with senior managers and IT managers involved in the implementation of the Vanguard, to explore perceptions and experiences of the programmes’ processes, outcomes and impact. Transcribed interview data and fieldwork notes were analysed using thematic analysis to generate category systems and repeated themes from a regional perspective. | Possible patient duplication in data set which could have occurred if they were sent to Northumberland Specialist Emergency Care Hospital after visiting an urgent care centre.  Analysis does not address operational issues relating to the roll out of the Vanguard, including redirection of patients to other services for limited periods.  Analysis focuses on Northumberland CCG population only and is not representative of total activity at Northumberland Specialist Emergency Care Hospital.  Potential seasonal effects not adjusted for.  Unable to assess full economic impact of the Vanguard. |
| Mid Nottinghamshire  Capita Transformation;  School of Health and Related Research, University of Sheffield.  (4 Reports) | Phase 1 Evidence review of urgent and emergency care (UEC) interventions. Quantitative effectiveness measured using UEC Care Channel shift tool (developed by Capita for the UEC Vanguards), statistical analysis using GRETl (Horizon model) to assess the impact on attendances, admissions and bed days, CUSUM used to test of the stability of the relationship between the national and local trends. Economic modally using UEC tool to assess the productivity of interventions and hence their return on investment.  Qualitative focused on experience of clinical pathways for urgent and proactive care for long term conditions - two focus groups with representatives from across all the Local Integrated Care Teams in Mansfield and Ashfield and Newark and Sherwood (n=? participants). Twenty telephone interviews with 9 NHS managers, 3 Social Services Managers and 8 GPs from across both CCGs. Observations of 2 MDT meetings.  Phase 2 Continuation of quantitative analysis from Phase 1 involving capacity modelling to develop scenarios around acute and community bed capacity and associated costs. Further cost benefit modelling using the UEC model.  Patient experience survey distributed by post to patients identified by Nottinghamshire Healthcare NHS Foundation Trust who had participated in the ‘Better Together’ programme within the last 12 months (n=837) | Phase 1 work presented in a ppt consultancy report with limited detail on methods. Evidence review and qualitative elements poorly reported.  One of the few sets of reports not to highlight any evaluation challenges.  Phase 2 patient survey yielded 17% response rate (n=143) |
| Salford | No Local evaluation commissioned via the new care models programme | N/A |
| South Somerset Symphony  South West Academic Health Science Network (SW AHSN),  South Central and West Commissioning Support Unit (SCW),  Centre for Health Economics, University of York  (1 Report) | Quantitative analysis using difference-in-differences with match controls to assess the evaluation of both Complex Care Teams and Enhanced Primary Care against key performance metrics (A&E Attendances, Emergency Admissions, Emergency Bed Days / Excess Bed Days / Length of Stay, Outpatient Appointments)  Data derived from Symphony dataset comprising information about each anonymised individual in the Somerset population for 2013-14 (577,982 individuals) and 2014-15 (583,618), used to provide baseline information and explore matching options. Planned to exploit other datasets to which SCW had access.  Qualitative SW AHSN to undertake survey work with 18 GP practices in the Enhanced Primary Care intervention. Validated questionnaires administered to a cohort of up to 400 (based on 100 new per month) new patients by health coaches to measure mental wellbeing (Warwick Edinburgh Mental Wellbeing Scale) and isolation (De Jong Loneliness Scale). Collation and analysis of the results of the above questionnaires as well as existing patient activation measures.  With existing EPC service, two focus groups of 8 to 10 patients from across localities, a focus group with 8 to 10 Health Coaches and face to face (or telephone) interviews with GPs (2 from each of the 4 localities) to understand views and gather learning about the programme to date. Patients were to be recruited via practices. | Significant data access issues. Agreement to share data with Evaluation team was not approved by the NHS Digital IGUARD panel until 23rd March 2017, nine months into the project. Therefore, CHE unable to undertake quantitative analysis within the original timeframe.  Qualitative work originally to be undertaken by SCW team. However, switched to SW AHSN start delayed of this work until April 2017, 10 months into project. At time of report no work had been undertaken. |
| Wirral | No Local evaluation commissioned via the new care models programme | N/A |
