# Supplementary material for: Investigating the nature and quality of locally commissioned evaluations of the NHS Vanguard programme: an evidence synthesis
Source: Health Res Policy Syst. 2021 Apr 12;19:63. doi: 10.1186/s12961-021-00711-3 (PMC8042862; doi:10.1186/s12961-021-00711-3)
Supplement: Supplementary file 3 — Additional file 3. Nature of local evaluations for MCP Vanguards [file 12961_2021_711_MOESM3_ESM.docx]

**Additional File 3 - Nature of local evaluations for the MCP Vanguards**

| Vanguard / Evaluator | Design | Limitations |
| --- | --- | --- |
| Better Local Care (South Hampshire)  RSM  PACEC Limited  (7 Reports) | Mixed methods evaluation using a ‘utilisation-focused’ approach including: a desk based literature review; analysis of publicly available secondary datasets to establish baseline levels for indicators; analysis of routine monitoring and feedback data collected by Better Local Care / Southern Health delivery teams; n=20 semi-structured telephone interviews with GPs, MCP delivery group members, project leads and other strategic stakeholders conducted between November and early December 2016 (n=18 follow up interviews conducted in March and April 2017); on-line survey of Better Local Care and wider Southern Health delivery teams (n=107/ estimated 250+ respondents across projects - targeted n=175-215 responses).  N=5 ‘deep dive’ case studies on the SDAS, One Team, WebGP, MSK, Paramedic projects. These included telephone interviews with patients using EQ-5D questionnaire (where access provided – patient numbers only presented for Eastleigh Frailty Clinic n=15).  Survey conducted pre- post Making Every Contact Count (MECC) training offered (n=15/21 responses from training recipients) N=4 interviews with health sector staff involved in delivering MECC training. | Significant data sharing and Information Governance issues. A lack of consistency in data availability and quality between projects. Local GDPR interpretation meant SUS data unavailable to the RSM team. Cost-savings analysis was hindered by the lack of a common standard on the unit cost of care by profession.  No details available on methods for desk based literature review.  Limited reporting of methods across all of the qualitative elements conducted. Difficult to establish numbers or respondents. |
| Birmingham and Sandwell (Connected Care Partnership)  Health Services Management Centre, University of Birmingham  (2 Reports) | Conceived as formative mixed methods evaluation: Interviews with (n=18) key informants involved in development of Vanguard and/or in the new care models programme nationally. Local sample included at least one representative from each of organisations that have formed the Partnership, the programme’s board and Steering Group, members of the programme team, commissioners and wider system stakeholders. Documentary analysis including the business case, logic models, minutes of partnership board and programme steering group meetings, detailed service specifications and quarterly reports of performance against local and national metrics – and reviews of published literature on key topics (on evidence about integrated care). Workshop with n=? key stakeholders, held March 2017, to explore options for economic evaluation.  Later phase of work included online survey to explore staff views and experiences of the changes being made (n=164/400 responses) a  partners survey (n=30/90 responses); interviews with (n=8) and focus groups (3 groups / 20 participants) with staff involved in specialist services, EPC and the clinical contact centre. | Limited details on documentary analysis and reviews of published literature.  Formative evaluation sets out a framework for evaluating Connected Care, including an assessment of the programme’s economic outcomes. Unclear what recommendations were actually taken forward. |
| Calderdale | No local evaluation commissioned | N/A |
| Dudley  The Strategy Unit (formerly Midlands and Lancashire CSU)  ICF Consulting Services Limited  Health Services Management Centre, University of Birmingham  (9 Reports) | Strategic level evaluation involved semi-structured interviews with n=21 local strategic stakeholders. Interviews were conducted between September and November 2017 by phone (n=12) and face-to-face (n=9). Interviews were recorded, transcribed and analysed thematically using NVivo 11 and a coding framework derived from Strategy Unit work (funded by NIHR) to synthesise the evidence for MCPs. An earlier round of interviews with n=16 stakeholders (14 of whom are interviewed later) were conducted by phone (n=7) and face-to-face (n=9) between June and July 2016.  Quantitative activity monitoring (undertaken quarterly?) on series of performance measures for Vanguard using time series (incorporating statistical process control) and funnel plots to assess variation in performance. Data sets from  April 2012 to December2017.  MDT evaluation included: literature review of MDTs; n=12 MDT meeting observations at four selected GP practices (3 observations per GP practice), supplemented by n=24 telephone interviews conducted with staff who attend MDT meetings; n=11 MDT stakeholder telephone interviews with local stakeholders from Dudley’s MDT Implementation Group; online survey with all MDT staff operating within Dudley’s 46 GP practices (n= 140 responses); n=7 telephone interviews with MDT patients. Quantitative data analysis on data provided by Dudley CCG on patients registered with GP practice MDTs and data from routine (SUS) datasets to explore the functioning of the MDT model and its potential impacts (trends in non-elective admissions and resulting length of stay).  Long Term Conditions Framework (LTCF) evaluation (Jan to March 2016) included initial online staff survey (n=55 responses from 36 GP practices); interviews with n=29 staff (GPs, practice managers, nurses and HCAs) on framework implementation; n=46 patient interviews/ observations of experience of patient centred consultations; review of n=271 (target n=350) care plans focused on the language and content of the goals set, and the extent to which these appeared patient-centred.  PROMs and PREMs evaluation included n=5 interviews with clinical and admin staff (n=5) to gather views on the language and suitability of measures, barriers and enablers for implementation and learning for the future; n=5 interviews with patients who had completed the measures; postal patient survey (n=17 responses).  Patient Activation Measure (PAM) evaluation included n=2 interviews with clinical staff and n=5 patients to judge the practicalities and acceptability of the PAM in practice. Limited activity analysis (based on n=167 who had their PAM level calculated) and a case study of a single patient. | Overarching evaluation questions but no integration / synthesis of data across projects; rather a set of discrete small scale evaluations undertaken.  Although strategic level interviews conducted at two time points, no attempt made to present an overarching analysis of change in perceptions over time.  Limited reporting of quantitative and qualitative elements of MDT evaluation; no denominator for staff survey and small number of patients. No details of literature review but assumption that this was synthesis (funded by NIHR) undertaken by Strategy Unit.  Very limited reporting of PROMs and PREMs evaluation; no denominator for postal survey and small number of staff and patients interviewed.  Very limited reporting of PAM evaluation; small number of staff and patients interviewed. |
| Encompass  Centre for Health Services Studies, University of Kent  (3 Reports) | Planned in two phases, utilising the iterative ‘Evidence Integration Triangle’ proposed by Glasgow (2013). Involves responsive mixed methods and use of a multi-agency steering group to agree methods and discuss emergent findings according to the pace of implementation.  The initial phase focused on four CHOCs but revised after 6 months due to slower than anticipated roll out and low patient recruitment. Two of the most ‘mature’ CHOCs became the central focus of the evaluation  Quantitative data collection included demographic data from service users interviewed (n=13); Interprofessional Collaboration Scale administered to staff before (n=24) and after (n=22) the evaluation period; Encompass activity metrics: CHOC service user profile (n=1,747), referrals, emergency admissions, bed days, length of stay on caseload, A&E attendances, staff type attending CHOCs, investment and cost savings. Analysis of impact on local healthcare services was based on a sample of 100 service users that were seen in the CHOCs between October 2016 and March 2017. Return on investment analysis based on the reduction in the number of emergency admissions multiplied by an average admission cost (derived from the prior admissions associated with the sample of 100 service users).  Data from 10 care plans from interviewed participants would be analysed per CHOC (n=20).  Qualitative data included semi structured interviews with service users (n=13) and professionals and senior managers (n=22); Four consecutive observations of team meetings across three CHOCs were also conducted. Qualitative interview data, including field notes were contextually and thematically analysed, grouped into relevant themes to provide an explanatory account.  Overarching analysis that triangulated data through blending and comparing the data sources within the research questions was conducted.  Evaluability assessment of Encompass social prescribing service provided by Red Zebra also undertaken. | Challenge to recruit service users with target levels of participation not met and resulting data imbalance between patient and professional sources. Difficult to engage professional staff to help identify service users and or participate in the evaluation themselves.  Some indication of data sharing and Information Governance issues as unable to link service user level data to analyse the impact of the CHOCs.  Absence of care plan data meant planned analysis not undertaken. (only n=1/13 individuals who were interviewed had care plan document in place and were aware of it and its purpose).  Although stated overall research design based on CFIR, no explicit engagement with framework beyond methods. Explicit use of ‘Evidence Integration Triangle’ throughout however. |
| Erewash  Cordis Bright  Peter Stone Consulting Limited  Economics Foundation  (4 Reports) | Cordis Bright baseline evaluation included: documentary analysis of strategic, operational and performance management information provided by the Vanguard (included the business case, activity and delivery plans, and financial data); a rapid evidence review of ‘what works’ in delivering similar integrated health and social care programmes; in-depth interviews with n=11 key Vanguard stakeholders identified by the evaluation steering group.  Peter Stone evaluation conducted in two phases; the first looking at the degree to which 4 projects had met any agreed outputs and outcomes. The second assessing the social and financial impact of each project (using SRoI analysis). Phase 1 data collection included facilitated discussions at project meetings supplemented by electronic surveys to gather a wider set of views around performance and delivery (planned follow up with telephone or email discussions of the impact of the projects and to gather data to support calculation of impact).  Economics Foundation evaluation included semi-structured interviews with n=15 people involved in the Vanguard; network mapping (undertaken during a workshop in Erewash), rapid review of similar workplace health and wellbeing programme; observations at workshops and local events. Some information also drawn from SRoI analysis completed by Peter Stone. | No final evaluation report from Cordis Bright available at time of this review.  Planned to interview members of the public who have had interactions with services provided by Vanguard. No participants were available to take part in an interview during the baseline phase.  Cordis Bright rapid review limited to search of Google Scholar and JSTOR. Looked at first 50 articles for each of 306 search terms. No other details of methods provided.  Very limited reporting of phase 1 data collection for Peter Stone evaluation; unclear how many interviews/ discussions / surveys sent. For SRoI, analysis not planned at outset so no pre-defined baseline and ‘less direct’ assessments used to give an overall impression of project impact.  Very limited reporting of all elements of Economics Foundation evaluation; no details on network analysis or rapid review. |
| Fylde Coast | No evaluation reports available at time of this review | Unknown |
| Lakeside Healthcare Northamptonshire | No local evaluation commissioned | N/A |
| South Notts (Principia)  Capita  (1 Report) | Phase 1 qualitative scoping interviews with n=12? Key informants, focus group (n=2), observation of meetings and presentations. Documentary analysis of value proposition, programme and project plans and documentation relating to the LTC and elective pathway work streams  Quantitative data sets reviewed included emergency and urgent care activity, financial and activity model of value proposition, QIPP savings tracker, Dr Foster? and Right Care? dashboards  unspecified data sources for initiatives in the LTC and elective pathway work streams.  Phase 2 evaluation intended as mixed methods with quantitative analysis using GRETl (Horizon model developed by Capita for the UEC Vanguards). Patient experience survey also proposed. | Unclear how many phase 1 interviews conducted, or meetings and presentations observed.  Phase 1 work presented in a ppt consultancy report with very limited reporting of all elements.  No phase 2 evaluation report available. |
| Stockport  Cordis Bright  (0 Reports) | Stockport Together reported that they appointed Cordis Bright as their formal evaluation partner in summer 2017 but no evaluation funding made available via the new models of care programme. No evaluation reports available at time of this review. | Unknown |
| Sunderland  NE Vanguard evaluation:  Institute of Health and Society, Newcastle University /  Centre for Public Policy and Health, Durham University /  Health and Life Sciences, Northumberland University  Cordis Bright  (5 Reports) | Combined NE Vanguard evaluation mixed-methods design, combining qualitative and quantitative approaches, to provide contextual understanding of the organisational, technological and economic facilitators and barriers shaping the implementation of the Vanguards programme. Evaluation conducted in three phases: (1) in-depth review of local documentation, semi-structured interviews with key stakeholders involved in the implementation of each Vanguard to identify organisational and technological enablers and barriers; (2) quantitative analysis and economic evaluation; (3) overarching synthesis and identification of emerging key messages for shared learning.  Quantitative analysis was conducted on all non-elective admissions and length of stay for the Sunderland CCG population from April 2013 -January 2017 (33 monthly data points pre and 13 post). Data for 30-day re-admissions was available from April 2015 to December 2016. Data regarding non-elective admissions included associated month and year of admission and length of stay. Analysis utilised ITS and Cox’s regression in order to make inferences regarding outcomes  Cost analysis involved an estimate of resource use as a consequence of introducing the Vanguard – cost included staff time (e.g. GP support, community nursing, pharmacy, social workers, and administration), training, community and care home beds, and digital solutions.  11 interviews, conducted with senior managers and IT managers involved in the implementation of the Vanguard, to explore perceptions and experiences of the programmes’ processes, outcomes and impact. Transcribed interview data and fieldwork notes were analysed using thematic analysis to generate category systems and repeated themes from a regional perspective.  Cordis Bright baseline evaluation included a review of 36 strategic and operational documents provided by Vanguard. This included reviewing the business case for the programme and its main work streams, performance evaluation group notes, governance structures, and performance data. A rapid evidence review of ‘what works’ in integrated health and social care programmes. In-depth interviews with 13 key programme stakeholders from 8 organisations.  Cordis Bright 2017 evaluation built on baseline work and included documentary review of 130 strategic and operational documents provided by Vanguard. A rapid evidence review of review of the ‘principles of an MCP’. In-depth interviews with 18 key programme stakeholders from 9 organisations. Additional elements were review and analysis of performance data; ‘cost effectiveness analysis’ of Vanguard programme (actually estimate of resource use as a consequence of introducing the Vanguard); four work stream focused ‘deep-dives’ of activity. | Post-Vanguard data only available for 12 months; potential seasonal effects were not controlled for.  Changes to service provision within Sunderland City Hospitals likely to have impacted upon key metrics such as non-elective A&E admissions and length of stay. Ambulatory wards were created and possible that these admissions would have increased the total number of non-elective admissions.  Cordis Bright rapid reviews limited to search of Google Scholar and JSTOR. Beyond search terms, no other details of methods provided.  Cordis Bright unable to conduct anticipated number of interviews with staff, stakeholders, or patients and service users. Not possible to develop case studies reflecting impact of service user pathways. Data on the full cost of Vanguard  delivery unavailable for the evaluation. |
| Tower Hamlets  UCL (embedded researcher)  (7 Reports) | Social prescribing: Activity analysis of 2,270 referrals made between 1 December 2016 and 31 July 2017. Only non-identifiable data was shared with the evaluation team. Analysis of costs of delivering the scheme and ‘EMIS’ search to examine changes in primary care use. Six month before and after analysis of n=890 patients GP appointment use (reduced from 3,388 to 2,970).  Patient experience (n=37 from baseline data of n=173) measured with MYCAW (Measure Yourself Concerns and Wellbeing - a questionnaire designed for evaluating complementary therapies in cancer support).  Two surveys conducted in August and September 2017, one with referrers (n=183 from 35/37 GP practices) and the other with VCS organisations (n=47 from 46 organisations). Complemented with 5 interviews with GPs, practice nurses and health care assistants; group interview with 3 VCS representatives; group interview with 3 members of the Wellbeing in Tower Hamlets Forum; focus group with social prescribers (n=8) and managers (n=5); 6 interviews with Steering Group members  Three follow up surveys conducted post Making Every Contact Count (MECC) training offered (n=142/1076 responses from training recipients)  Locality based approaches evaluation included documentary analysis of contextual information pertaining to Vanguard (collated through negotiation and collaboration with relevant stakeholders from the programme) – used as basis for interview guides; n=20 semi-structured interviews with key stakeholders at strategic and operational levels of programme; 200 hours of observation of key board, steering workstream commissioning and monthly locality meetings; field notes of informal discussions also used as a source of data. | Very limited reporting of all social prescribing quantitative and qualitative elements; small numbers of participants and limited follow up of patients.  Unable to analyse GP appointment data on a borough-wide basis as originally planned.  Social prescribing activity and costs are benchmarked against findings from a non-systematic review with a high risk of bias (Polley et al 2017) |
| West Cheshire | No evaluation reports available at time of this review | Unknown |
| West Wakefield  North of England Commissioning Support Unit (NECS)  HealthWatch / Niche Health and Social Care Consulting  York Consulting  (4 Reports) | Qualitative component included semi-structured interviews were conducted with 23 individuals across 9 GP practices. Patient experience assessed via 720 patient surveys collected during the course of visits to GP practices by Healthwatch Wakefield.  Quantitative analysis involved building on metrics developed metrics and dashboards for the Vanguard for reporting to NHS England. Data included lengths of stay, admissions and A&E attendances, walk in centre attendance rates and referral counts. In addition the number of care navigations, physio and pharmacy time used were collected. A cost-consequence analysis was used to identify the associated costs and outcomes of the HealthPod intervention.  HealthWatch also conducted staff experience interviews with 35 staff (from the three Hubs) and their managers conducted in August/ September 2016. Thematic analysis used to identify the key themes arising from interviews.  Report also describes 680 interviews, 43% conducted with the carer present, with people who had been receiving Connecting Care services. These were conducted in 2014 so appears to be a pre Vanguard evaluation.  York Consulting conducted 56 one-to-one, semi-structured interviews with members of staff involved in Connecting Care. Staff interviewed were nominated by members of the evaluation steering group or by the operational leads for Connecting Care within the partner organisations. | Some indication of data sharing and Information Governance issues though unclear how detrimental these were.  Issues with recording of data in some GP practices, meaning all care navigation activity may not have been captured. Some GP practices did not take up invitation to take part in the evaluation  Lack of robust data collection and reporting processes for physio and pharmacy interventions. |
