# Supplementary material for: Investigating the nature and quality of locally commissioned evaluations of the NHS Vanguard programme: an evidence synthesis
Source: Health Res Policy Syst. 2021 Apr 12;19:63. doi: 10.1186/s12961-021-00711-3 (PMC8042862; doi:10.1186/s12961-021-00711-3)
Supplement: Supplementary file 4 — Additional file 4. Local evaluation questions [file 12961_2021_711_MOESM4_ESM.docx]

**Additional File 4 - Local evaluation questions**

**Table 1: Evaluation questions for the Enhanced Care Homes Vanguards**

| Vanguard | Local evaluation questions |
| --- | --- |
| Airedale | How does the model of telemedicine (TM) impact on care home utilisation?  Which care homes use TM and why?  Which care homes don’t use TM and why?  What role does TM model in place play?  What impact does TM utilisation have on key outcomes of healthcare utilisation?  A&E attendances, Non-elective admissions, conveyance, GP call outs  Is TM perceived by those closest to it as an appropriate, acceptable, effective healthcare delivery method?  What difference does the model of TM make?  What are the key benefits, challenges of TM?  How can TM in care homes be improved? |
| Gateshead | (From North East Vanguard evaluation)  What new conceptual understandings have been used to develop NCMs and what theories of change underpin them?  What new opportunities have become available with the five North East Vanguard Programmes? What challenges have participating organisations experienced in implementing the Vanguard Programmes and how have these challenges been addressed?  To what extent, and in what ways, has digital innovation shaped NCMs’ aspirations and achievements?  What impact has each Vanguard had on the efficiency of the local health and care economy?  (From pathways of care (PoC) qualitative evaluation)  How has the PoC developed and what are the keys to success?  What are participant views on the approach to clinical leadership and engagement?  How and why has the approach changed practice and influenced resident care?  How may the model and team need to change across different settings to enhance care delivery?  What are the main anticipated barriers to implementation?  (From Whzan Health System for recording NEWS evaluation)  Assess the relationship between NEWS score and frailty, cognitive impairment, dependency, functional ability and treatment outcomes in the acutely ill older care home resident indicators.  Explore care home staff, NHS community nurses working into care homes and GPs’ understanding of the factors that are key determinants of the presentation of acute illness in frail older people.  Explore care home staff, NHS community nurses working in to care homes and GPs’ views, experiences and barriers to the use of NEWS  Examine the impact of the introduction of NEWS on clinical decision-making process in relation to treatment of the acutely ill older care home resident.  (From University of Sunderland qualitative evaluation)  Explore key stakeholders’ perceptions of current ways of working as a result of the EHCH Vanguard implementation |
| East and North Hertfordshire | How the HomeFirst and Stroke ESD teams worked and the perceived strengths and weaknesses of the service?  What is the impact of the HomeFirst and Stroke ESD on the wider system,  What is the patient and carer experience of the service  What are the outcomes and value for money of the service? |
| Nottingham City | Programme evaluation questions:  What is the context (e.g. history, culture, relationships, health inequalities, local and national policies, national legislation) in Nottingham City into which the Care Homes Vanguard has been implemented?  What key changes has the vanguard made and who is being affected by them? How have these changes been implemented?  What is the change in resource use and cost for the specific interventions that encompass the new care model programme locally? How is the vanguard performing against its expectations and how can the care model be improved? Include the resource used by the programme team, stakeholders and partners.  What impact is the vanguard having on resident and workforce outcomes and experience, and the way in which resources are used in the local health system? This should be compared against a counterfactual in which the vanguard interventions have not been delivered. This includes:   - How much will it cost to be business as usual? - How much of the cost is covered by the savings generated? - How soon can it happen?   Are the components of the care model working and really making a difference?  What are the ‘active ingredients’ of the model of care? Which aspects, if replicated elsewhere, can be expected to give similar results and what contextual factors are prerequisites for success?  What are the unintended costs and consequences (positive or negative) associated with the new model of care on the local health economy and beyond?  Work stream specific evaluation questions:  New Technology  What learning has there been from implementing these interventions?  How effective are the elements of the Assistive Technology work stream in relation to:   - Reducing the number of care home residents requiring emergency admission or readmission to hospital or GP call out - Reducing the length of stay in hospital through awareness and confidence of Assistive Technology in the Care Home to support the care of the resident including at end of life - Producing a better understanding of who benefits from AT and how to better identify and target those groups in Care Homes - Staff utilisation and increasing the productivity of staff in Care Homes - A cost effective service and return on investment   Clinical Pharmacy  What learning has there been from implementing these interventions?  How effective are regular clinical pharmacy reviews in relation to:   - Cost savings: drug costs and time saved by other health care professionals i.e. increased productivity of the multi-disciplinary team - Cost avoidance: harm reduction through review of medication to reduce errors, adverse drug reactions and avoided hospital admissions - Improved systems and processes and governance associated with medicines particularly at the interfaces (i.e. from secondary care to care home and vice versa and interactions with primary care); and implementation of improved communications with GP practices. - Increase in appropriate prescribing and decrease in inappropriate prescribing in line with local and national guidance and the embedding of the principles of medicines optimisation for the frail elderly - Residents, carers and staff who are better informed and empowered to make choices about their medicines |
| Sutton | What is the context into which Sutton’s model has been implemented?  What key changes has the Sutton model made and who is being affected by them? How have these changes been implemented?  What is the change in resource use and cost for the specific interventions in the new care model? How is the Vanguard performing against its expectations and how can the care model be improved?  What impact is the Vanguard having on:  residents’ outcomes and experiences?  residents’ families and carers experiences?  the competence and confidence of care staff and managers in the care homes?  the workforce commissioned to support the care staff and managers in the care homes?  the way in which resources are used across the local health and social care system?  Which components of the care model are really making a difference? In particular, which key components of the model are making the biggest difference, and which of all the components are interdependent and independent?  What are the ‘active ingredients’ of the care model? Which aspects, if replicated elsewhere, can be expected to give similar results, and what contextual factors are prerequisites for success?  What are the unintended costs and consequences (positive or negative) associated with the new model of care on the local health and social care system, and in general? |
| Wakefield | Is care more coordinated and seamless?  Has the Vanguard reduced the demand on secondary care from Care Homes?  What impact has the Vanguard had on patient outcomes and experience? Including:  End of life care  Management of long term conditions and falls  What has been the impact on resident wellbeing?  Which elements of the intervention have had the greatest impact and could they be replicated elsewhere?  What has been the impact of involving community anchors in the lives of care home residents? |

**Table 2: Evaluation questions for the PACs Vanguards**

| Vanguard | Local evaluation questions |
| --- | --- |
| Harrogate | Work stream 1 (Qualitative Process & Theory-led evaluation)  What key changes have the Vanguards made and who is being affected by them?  How have these changes been implemented?  What are the ‘active ingredients’ of a care model?  Which aspects, if replicated elsewhere, can be expected to give similar results and what contextual factors are prerequisites for success?  Work stream 2 (Metrics Development)  To develop a way of identifying and utilising metrics to measure impact across the dimensions of quality, cost and activity;  To create a methodology that can be used into the future to enable the local system to continually evaluate the impact of the care model;  To develop a dashboard to visualise the data and support operational and quality monitoring;  To demonstrate the impact and to inform the development of the new care model.  Work stream 3 (Economic evaluation)  Assessment of the economic costs of the Integrated Response Service team  Analysis of the impact of the Integrated Response Service on key NHS resource use metrics. |
| North East Hampshire and Farnham | Three overarching aims for the programme of evaluation  To understand the patient, staff and system outcomes of the new models of care and how they were implemented  To work with the Vanguard to use the evaluation findings in further development of the programme  To share the learning from the evaluations to enable spread and adoption to other health care systems |
| Isle of Wight | Overarching questions not stated  Case Management of those at risk (CMoTAR) in primary care  What is the acceptability of this model?  What are the implementation issues associated with delivery of the CMoTAR programme?  What are the perceived effects of this approach to multi-disciplinary team (MDT) working on primary care and on people who receive care?  What are the challenges and how could the service be improved?  Local Area Coordinators  What is the nature, extent and impact of Local Area Coordination as part of the My Life a Full Life new care model?”  Care Navigators  No explicit questions stated for the evaluation of the Care Navigator Service |
| Morecambe Bay | Aims to ‘address the question set developed by the national NCM team’:  What is the context (e.g. history, culture, relationships, health inequalities, local and national policies, national legislation) in each Vanguard into which new care models have been implemented?  What key changes have the Vanguards made and who is being affected by them? How have these changes been implemented?  What is the change in resource use and cost for the specific interventions that encompass the new care models programme locally?  How are Vanguards performing against their expectations and how can the care model be improved?  What impact are the Vanguards having on patient outcomes and experience, the health of the local population and the way in which resources are used in the local health system?  Which components of the care model are really making a difference?  What are the ‘active ingredients’ of a care model? Which aspects, if replicated elsewhere, can be expected to give similar results and what contextual factors are prerequisites for success?  What are the unintended costs and consequences (positive or negative) associated with the new models of care on the local health economy and beyond? |
| Northumberland | (From North East Vanguard evaluation)  What new conceptual understandings have been used to develop NCMs and what theories of change underpin them?  What new opportunities have become available with the five North East Vanguard Programmes? What challenges have participating organisations experienced in implementing the Vanguard Programmes and how have these challenges been addressed?  To what extent, and in what ways, has digital innovation shaped NCMs’ aspirations and achievements?  What impact has each Vanguard had on the efficiency of the local health and care economy? |
| Mid Nottinghamshire | Aims to address the question set by NHS England:  What impact is the Vanguard having? This should be compared against the counter factual  What key changes has the Vanguard made and who is being affected by them?  How have these been implemented?  How is the Vanguard performing against expectation and how can the care model be improved?  Where are unintended cost and consequences (positive and negative) associated with the new models of care on the local health and social care economy and beyond  How do we identify impact of individual interventions when we are implementing multiple interventions in a programme?  What is the change in resource use and cost for specific interventions that encompass the new care models programmes locally?  How are costs shared between commissioners including both the NHS and Local Authorities and how can the output/outcomes be attributed to individual commissioners?  What is the context (e.g. culture history, relationships, health inequalities, local and national policies national legislation) of the Vanguard into which new models are being implemented?  What are the “active ingredients,” of the care model? Which aspects if replicated elsewhere, can be expected to give similar results and what contextual factors are prerequisites to success?  What impact is the Vanguard having on improving system resilience? |
| Salford | No local evaluation commissioned via the new care models programme |
| South Somerset Symphony | Is Symphony meeting the key outcome statements – is the programme seamless, person centred and well integrated?  The Symphony programme focuses on a sustainable model of primary care and has engaged staff to make it a better place to work. Is there evidence that this is the case?  The Symphony team would wish to understand the experience of delivering and receiving care under the Health Coach scheme (e.g. what is working best for them and why, what is not going so well. Which bits of what they do work best?)  It would be helpful to understand which bit of what Health Coaches do is most effective; who are the people for whom this approach is working and for whom it isn’t?  How the ‘communities worker’ model is benefitting the programme and how groups can evaluate how their projects are improving wellbeing e.g. social isolation?  Understand the experience of the extensivist  Understand the experience of a practice in delivering EPC  What elements of the programme are working well, and for those that aren’t what should be done instead?  What are the gaps? What haven’t we done that could be done in the programme?  Does the New Care Model ensure the health and care system is sustainable?  Is the wellbeing of patients being improved and sustained? |
| Wirral | No local evaluation commissioned via the new care models programme |

**Table 3 – Evaluation questions for the MCP Vanguards**

| Vanguard | Local evaluation questions |
| --- | --- |
| Calderdale | No local evaluation commissioned |
| Birmingham and Sandwell (Connected Care Partnership) | (from Phase 1 evaluation)  What key process changes has the Vanguard made and what changes is it struggling to make? How have these changes been implemented and who is being affected by them?  What impact is the Vanguard having on staff experience? How can it improve/measure team working and foster a sense of collective clinical ownership and accountability across our partners?  What impact is the Vanguard having on patient experience? How can it collect and use both patient experience and patient-reported outcomes to inform programme development, on an individual level, service level and system level?  What are the relative cost/benefits of individual programmes/resources and how can the Vanguard use this to refine the economics of the Connected Care programme?  What programmes should be scaled/expanded and or changed/stopped based on an economics appraisal? |
| Dudley | (Overarching evaluation questions)  What is the context for the programme?  What was the perceived need for change and why was an MCP model seen as an appropriate response?  What were the major changes initiated by the programme and how well were they implemented?  How was the programme experienced across the system, e.g. by: the public, patients, staff and stakeholders?  What outcomes were achieved by the programme? How were these outcomes achieved? Were there any negative or unintended consequences of the programme?  In what ways and to what extent has the programme changed / improved the pattern of resource use within the local health economy?  To what extent has the programme addressed its founding rationale?  What lessons - for practice and policy - can be derived from Dudley’s experience?  What would need to be considered in order to replicate component parts? Conversely, what can Dudley learn from analogous practice elsewhere?  (from the MDT evaluation)  What is an MDT, which services are represented within Dudley MDTs and what models operate in Dudley?  How have Dudley MDTs been developed to date, why were they set up and what problems or opportunities were they established to address?  How were the MDTs intended to operate and how are they operating in practice?  What factors facilitate working in a multidisciplinary way and what barriers exist that hinder this way of working?  What difference do Dudley’s MDTs have on patients and local services and how is this difference achieved?  How should Dudley MDTs develop in the future and what lessons can be drawn from the experience in Dudley to date? And,  What measures could be used by the MDTs themselves to establish whether they are having the desired effect? |
| Encompass | What impact are the Community Hub Operating Centres having on user outcomes and experience? (eg health and wellness, seamless care, access to resources, self-management and independence at home)  What are the components of the care model delivery (or ‘active/successful ingredients’) that are really making a difference? (eg associated with leadership, collaboration, continuity of care, nature of change, working environment, information sharing, workforce change)  What are the influencing contextual factors and how have they affected implementation and outcomes? (e.g. history, culture, relationships, working arrangements, contracts, local and national policies)  What changes to the use of resources and activity in the local health system have taken place and to what costs? (eg costs for specific new care model interventions, hospital use, intended and unintended costs)  What could be improved, replicated and sustained? |
| Erewash | (from Cordis Bright baseline evaluation)  What is the context (e.g. history, culture, relationships, health inequalities, local and national policies, national legislation) in the MCP into which new care models have been implemented?  What key changes have been made in Erewash and who is being affected by them? For example, is there a particular patient demographic being affected by the new service provision? How have these changes been implemented?  What is the change in resource use and cost for the specific interventions that encompass the new care models programme locally? How is the MCP performing against its expectations and how can the care model be improved?  What impact is the MCP having on patient outcomes and experience, the health of the local population and the way in which resources are used in the local health system? This should be compared against a counterfactual in which the Vanguard interventions have not been delivered.  Elicit staff views regarding the on-day service. Are there any changes that could be made to the current delivery model?  Which components of the care model are really making a difference?  What are the ‘active ingredients’ of a care model? Which aspects, if replicated elsewhere, can be expected to give similar results and what contextual factors are prerequisites for success?  What are the unintended costs and consequences (positive or negative) associated with the new models of care on the local health economy and beyond?  (from the Peter Stone evaluation)  To produce an evaluation of four programmes funded by Wellbeing Erewash with a view to determining if they had delivered agreed outputs and outcomes and delivered social and financial value  (from the Economics Foundation evaluation)  To what extent has Wellbeing Erewash supported people in Erewash to come together, increase their understanding of health and wellbeing and take action as part of a social movement? What difference has the project made, how and for whom? |
| Fylde Coast | No details available |
| Lakeside Healthcare Northamptonshire | No local evaluation commissioned |
| South Hampshire | Understand the mechanisms that have led to changes in outcomes, including an analysis of factors that underpin the impact and replicability of the models of care.  Evaluate performance of the programme against stated aims and objectives, incorporating national and local metrics, routine health service data, as well as analysis of locally collated quantitative and qualitative data.  Undertake an in-depth evaluation of the main models of care, incorporating but not limited to the outputs and outcomes in the programme and thematic logic models.  Evaluate the enablers and barriers to organisational change at primary care and locality level.  Evaluate the impact of the Better Local Care programme from a systems, financial and broader economic perspective. |
| South Notts (Principia) | (from Phase 1 evaluation)  How comprehensive is the MCP's logic model: To what extent are inputs (financial and people), activities, outputs and outcomes mapped, understood and quantified?  To what extent is the combined/aggregated impact of interventions understood?  Does the MCP have a consolidated view of when interventions are coming on stream and when their impact is anticipated?  How is the MCP measuring and tracking inputs, activities, outputs and outcomes? Are there any gaps in what is currently being collected, measured and reported? Are there any concerns around data quality?  Can the MCP provide a baseline position? Are there any gaps?  To what extent are anticipated savings, shifts out of hospital and return on investment grounded in evidence? What assumptions have been made and are they reasonable?  What factors make Rushcliffe unique from or comparable to other areas and what are the implications of this for transferability?  What information is the MCP using to target interventions effectively?  What are the early indications for the impact of interventions? |
| Stockport | (from evaluation tender specification)  Compared to the traditional system/service, is the new integrated service solution better for the patient?  How does the new patient journey differ? Does it work for the majority?  In what way are the 8 neighbourhoods different in terms of pathway, outcome, cost, access to borough wide services, acute interface and healthy community initiatives?  Are some neighbourhoods more successful than others? What are the characteristics associated with successful outcomes?  Have patient level outcomes improved across the borough/by neighbourhood?  How satisfied are people and carers? Does this differ between neighbourhoods or parts of the system?  Do staff think the new system is an improvement on the traditional? |
| Sunderland | (From North East Vanguard evaluation)  What new conceptual understandings have been used to develop NCMs and what theories of change underpin them?  What new opportunities have become available with the five North East Vanguard Programmes? What challenges have participating organisations experienced in implementing the Vanguard Programmes and how have these challenges been addressed?  To what extent, and in what ways, has digital innovation shaped NCMs’ aspirations and achievements?  What impact has each Vanguard had on the efficiency of the local health and care economy?  (From Cordis Bright baseline evaluation)  Consider the context of the Sunderland Care model, including Sunderland specific approaches to MCP development.  Review the Recovery at Home/Older People’s Assessment and Liaison service, focussing upon the model of service operation, outcomes for patients and partners, longer term sustainability, return on investment (ROI) and replicability.  Review the Community Integrated Teams, including the roles of Living Well Link Workers, MDT Co-ordinators, and the clinical leadership function of GPs. Consideration of outcomes for partners and patients, longer term sustainability, return on investment (ROI) and replicability. Consider how effectively the teams have integrated.  Review the programme of Enhanced Primary Care, and the interventions that form part of this. Consideration of the impact of these projects in supporting patients with long term conditions, as well as the ability to release capacity for General Practice  Consider the leadership and governance functions specific to Sunderland, including the CCG assurance function, Integrated Community Services Provider Board, and Programme Management Office.  Review the overall outcomes of the Vanguard programme, performance against expectations, and any unintended outcomes.  (From Cordis Bright 2017 evaluation)  The impact of ATB on outcomes for:  Patients/service users and relatives/families;  Health and social care staff involved in/with ATB across Sunderland;  The wider health and social care system across Sunderland  Economic impact;  Process factors regarding the implementation and operation of the ATB Programme;  The overall impact the programme has had on patients, service users, residents, health and social care staff and the health and social care system in Sunderland. |
| Tower Hamlets Together | (from social prescribing evaluation)  To explore the extent to which the programme has embedded itself within the Tower Hamlets primary care system, its impact on the range of services available to users and the impact on those services.  To assess the improvement in health and wellbeing of users of the social prescribing programme.  To understand the experience of referring into and delivering the programme.  To assess the extent to which social prescribing can facilitate community development in terms of connecting residents with each other for support.  To establish the cost savings of the programme within the context of health care and wider public sector budgets  To recommend an ideal social prescribing model, including level of funding required  (from the locality based approaches to integrated care evaluation)  To explore the effectiveness of the CHS model to enable collaborative working across the partnership at the strategic, operational and service delivery levels and its perceived impact on staff and person outcomes.  To establish how the implementation of the Vanguard programme and CHS model has engaged front line practitioners and service users in terms of challenging their values and norms and changing their behaviours.  To assess the effectiveness of the Vanguard programme and CHS model in involving and engaging service users and citizens in programme activities and in their experience of using THT services. |
| West Wakefield | (from NECS interim report)  How would you describe the context of the programme/population including an understanding of history, cultures, relationships, health inequalities, local and national policies and national legislation?  What key changes have the Vanguards made and who is being affected by them? How have these changes been implemented?  What is the change in resource use and cost for the specific interventions that encompass the new care models programme locally? How is the Vanguard performing against their expectations and how can the care model be improved?  What impact is the Vanguard having on patient outcomes and experience, the health of the local population and way in which resources are used in the local health system?  Which components of the care model are really making a difference?  What are the ‘active ingredients’ of the care model? Which aspects, if replicated elsewhere, can be expected to give similar results and what contextual factors are prerequisites for success?  What are the unintended costs and consequences (positive or negative) associated with the new care model on the local health economy and beyond?  (from York Consulting report)  To obtain feedback on the implementation and early outcomes of Connecting Care from staff that are involved in its delivery |
